# 

**Published:** 1894-12

**Authors:** 


### CONTENTS.

*EDITORIAL.*

PAGE

Reducing Temperatures in Fever, . . . . . . . . . . 379

*MISCELLANEOUS.*

A Bird's-Eye View of Bovista. C. C. Smith, M. D., . . . . . . . . . . 381

Clinical Experience with the Single Dose of the CM Potency. E. E. Case, M. D., . 383

Homœopathic Medical College of Missouri, . . . . . . . . . . 388

Neuralgia in the Teeth. Thos. C. Bunting, M. D., . . . . . . . . . . 390

In Memoriam—J. P. Dake, M. D., . . . . . . . . . . 390

Prof. J. P. Dake, M. D.,—Personal Recollections. Bushrod W. James, M. D., . . . . 392

The Organon and Materia Medica Club of the Bay Cities of California, . . . . . . 397

An Antidote to Cocaine. J. G. Gundlach, M. D., . . . . . . . . . . 402

An Involuntary Proving of the Fruit Wasp. C. F. Menninger, M. D., . . . . . . . . 403

The Physician's Library. E. Cranch, M. D., . . . . . . . . . . 406

Waiting Upon the Remedy. F. Preston, M. D., . . . . . . . . . . 409

A Case of Diphtheria Treated with Antitoxin. Arnold W. Catlin, M. D., . . . . . 410

Scarlatina and Variola. Translated by Frederick Preston, M. D., . . . . . . . . . 413

American Institute of Homœopathy. George B. Peck, M. D., . . . . . . . . . . 414

Death of Alexander III, and His Physicians, . . . . . . . . . . 415

*BOOK NOTICES AND REVIEWS.*

A Practical Treatise on the Diseases of the Hair and Scalp, . . . . . . . . . . 417

A Synopsis of the Practice of Medicine, . . . . . . . . . . 418

The Physician's Visiting List, . . . . . . . . . . 419

Health Culture, . . . . . . . . . . 419

The Scientific American, . . . . . . . . . . 419

The Philanthropic Index and Review, . . . . . . . . . . 419

The Standard Dictionary of Funk & Wagnalls, . . . . . . . . . . 420

*NOTES AND NOTICES.*

The Metropolitan Post-graduate School—The Late Dr. L. B. Wells—The Homœopathic Society of Chicago—Bile Pigment—Morbid Conditions of the Heart, . 420

Fun for Doctors, . . . . . . . . . . 422

